# Image Analysis and Untargeted Metabolomics Reveal Potential Phytotoxins from *Fusarium venenatum* Against Major Parasitic Weed *Phelipanche ramosa* (L.) Pomel

**DOI:** 10.3390/toxins16120531

**Published:** 2024-12-10

**Authors:** Ana Bendejacq-Seychelles, Lisa Martinez, Anaïs Corréard, Jean Chrisologue Totozafy, Christian Steinberg, Jean-Bernard Pouvreau, Carole Reibel, Grégory Mouille, Samuel Mondy, Lucie Poulin, Stéphanie Gibot-Leclerc

**Affiliations:** 1 Agroecologie, INRAE, Institut Agro Dijon, Université Bourgogne Franche-Comté, 21000 Dijon, France; ana.bendejacq-seychelles@inrae.fr (A.B.-S.); christian.steinberg@inrae.fr (C.S.); carole.reibel@agrosupdijon.fr (C.R.); samuel.mondy@inrae.fr (S.M.); 2 UMR 6286, CNRS, US2B, Nantes Université, F-44000 Nantes, France; lisa.martinez@etu.univ-nantes.fr (L.M.); jean-bernard.pouvreau@univ-nantes.fr (J.-B.P.); lucie.poulin@univ-nantes.fr (L.P.); 3 Institut Jean-Pierre Bourgin, INRAE, AgroParisTech, Université Paris-Saclay, 78026 Versailles, France; jean-chrisologue.totozafy@inrae.fr (J.C.T.); gregory.mouille@inrae.fr (G.M.)

**Keywords:** *Fusarium venenatum*, *Phelipanche ramosa*, fungal metabolites, image analysis, untargeted metabolomics, phytotoxicity

## Abstract

Branched broomrape (*Phelipanche ramosa* (L.) Pomel), an obligate parasitic weed with a wide host range, is known for its devasting effects on many crops worldwide. Soil fungi, notably *Fusarium* sp., are described as pathogenic to broomrape, while the hypothesis of the phytotoxicity of fusaric acid produced by *F. verticillioides* for parasitic weeds of the genus *Orobanche* has been proposed. Using image analysis and untargeted metabolomics, this study investigated fungal metabolites phytotoxic for *P. ramosa* and produced by the *F. venenatum* MIAE02836 strain, isolated from symptomatic broomrapes and identified as a promising candidate for broomrape biocontrol. Phytotoxicity tests of crude extracts from the fungus alone or in interaction with broomrape on *P. ramosa* microcalli and quantification of necrosis by image analysis confirmed the phytotoxic potential of *F. venenatum* MIAE02836 metabolites towards the early developmental stages of *P. ramosa*. Data analysis of a non-targeted metabolomics approach revealed numerous metabolites produced by *F. venenatum* MIAE02836. Four of them, accumulated during interaction with the parasitic plant, are known for their phytotoxic potential: maculosin, cyclo(Leu-Phe), phenylalanyl-D-histidine and anguidine. These results suggest that combining image acquisition of the microcalli screening test and untargeted metabolomic approach is an interesting and relevant method to characterize phytotoxic fungal metabolites.

## 1. Introduction

Branched broomrape (*Phelipanche ramosa* (L.) Pomel) is a holoparasitic plant known for its devastating effects on crops with economic importance [1]. In France, for instance, branched broomrape mainly affects winter oilseed rape and tobacco, where it can cause up to 90% yield losses [2,3]. Despite the major impact of *P. ramosa* on crop yields, there is, to date, no effective curative method among the traditional ones (herbicides, crop rotation, varietal breeding). This can be explained by the distinguishing features of *P. ramosa,* especially its life cycle with an extended underground phase, its strict dependence on a host and its host preference, reflected by the presence of various genetic clusters or pathovars, and its important reproductive capability [4,5,6]. However, these features also make this parasite a compelling target for biocontrol approaches [4,5]. 

Fungi are thought to be a promising avenue for biocontrol of *P. ramosa* [7,8,9,10,11]. Different strains of fungi, often belonging to the *Fusarium* genus, were isolated on the basis of symptoms they caused on broomrape in a rather reproducible manner under controlled conditions. Their inoculation in the field, despite varied formulations, did not provide the expected results [7,12]. The outcome of fungal isolates and broomrape interactions is determined by the invasive mechanisms implemented by the former and the defense reaction ones activated by the latter, the expression of both mechanisms depending on environmental conditions. Unfortunately, these mechanisms have not been studied precisely in the context of *Fusarium*–broomrape interactions. The hypothesis of metabolites such as fusaric acid has been proposed to explain the herbicidal activity of *F. verticillioides* against *Orobanche* spp. [13]. We propose to explore this hypothesis regarding a strain of *F. venenatum* (MIAE02836), recently identified as a potential mycoherbicide for *P. ramosa* parasitizing tobacco and oilseed rape [11]. This *F. venenatum* strain is differentiated by its ability to inhibit seed germination and necrotize stems of *P. ramosa* without being pathogenic to the host plant tested (tobacco) [11]. This dual competence makes it an ideal candidate to know if its herbicidal activity is linked to the production of specific metabolites upon interaction with *P. ramosa* pathovars. *F. venenatum* has a reputation for being non-pathogenic and has been used for decades as a source of mycoproteins [14,15,16,17]. However, this fungi is also able to produce mycotoxins [18,19,20,21] and has been recently characterized as pathogenic to wheat [22] and to potatoes [23]; hence, the interest in searching phytotoxic fungal metabolites supposed to be more eco-friendly, biodegradable and efficient to combat herbicidal resistance [24]. Using them for biocontrol instead of fungal strains will avoid toxicity to non-target plants or organisms by mycotoxins.

To date, tests of fungal metabolites or suspension on *P. ramosa* have mainly been carried out in Petri dishes for germination and co-culture for plant development [25,26,27,28]. Due to the microscopic size of *P. ramosa* seeds, the usual Petri dish tests, with counting under a binocular magnifying glass, are long, tedious, and unsuitable for large sampling plans. Co-cultures take a long time to set up and reveal the complexity of the tripartite relationship between the parasitic plant, the host plant, and the pathogen [2,28]. The culture of broomrape calli, developed by Fernández-Aparicio et al. on *P. aegyptiaca*, is an innovative method [29]. The adaptation of this culture system in 96-well plates [30,31,32] offers interesting features: (i) cultivation of the parasitic plant in the absence of the host is made possible by the use of a sucrose-concentrated culture medium; (ii) production is rapid (three to four weeks) and in a sterile environment; (iii) a large number of samples are available (96 wells per plate), which facilitates metabolite screening during phytotoxicity tests. Fungal filtrate-induced necrosis can be observed under the microscope, but the analysis remains mainly qualitative. Computerized image analysis can bypass most of the biases due to human observation and has become an essential tool in disciplines such as biology [33]. Recently, a high-throughput phenotyping tool for seed germination in 96-well plates was developed [34]. Image acquisition from the wells was automated, and the transferred data were analyzed by machine learning using sprouted seed scoring, which augured thousands of samples to be explored accurately, rapidly, and repeatedly.

Searching for phytotoxic fungal metabolites relies on a biology-guides approach coupled with untargeted metabolomics. Crude fungal metabolite filtrates are tested for their potential phytotoxic activity and analyzed by liquid chromatography coupled with a mass spectrometer. Characterization of metabolites is usually performed by molecular networks and searching in natural products databases based on tandem mass spectrometry (MS/MS) spectra and exact mass [24]. 

So, the present study aims to identify *F. venenatum* MIAE02836 metabolites having a phytotoxic activity toward *P. ramosa*. Achieving this objective involves testing two hypotheses: (i) the phytotoxicity of the metabolites can be evaluated by a test on broomrape microcalli using image analysis to quantify necrosis induced by fungal filtrates; (ii) untargeted metabolomics data analysis will identify fungal metabolites of interest., A dual approach using image analysis and a non-targeted metabolomics analysis was therefore implemented to test these hypotheses and achieve our objective.

## 2. Results

### 2.1. Necrotic Activity of Fusarium venenatum Filtrates on Broomrape Microcalli

The necrotic activity of filtrates of *F. venenatum* MIAE02836 in interaction with broomrape (modality A) or alone (modality B) was evaluated on *P. ramosa* microcalli by image analysis on four of the six days of incubation (D0, D1, D5 and D6 after addition). Necrotic activity was qualitatively defined by the apparition of brown hues on healthy-white microcalli. Microcalli from negative control (sterile distilled water) remained predominantly white and healthy until D6. Slight necrosis appeared around D5 due to the absence of nutrients in the wells. A change in microcalli coloration (light brown hue) was observed as early as D1 for modalities A and B and continued progressively over time. The change in coloration was more rapid and pronounced on microcalli treated with filtrates from modality A, suggesting greater necrotic activity of these extracts. 

**Figure 1:**
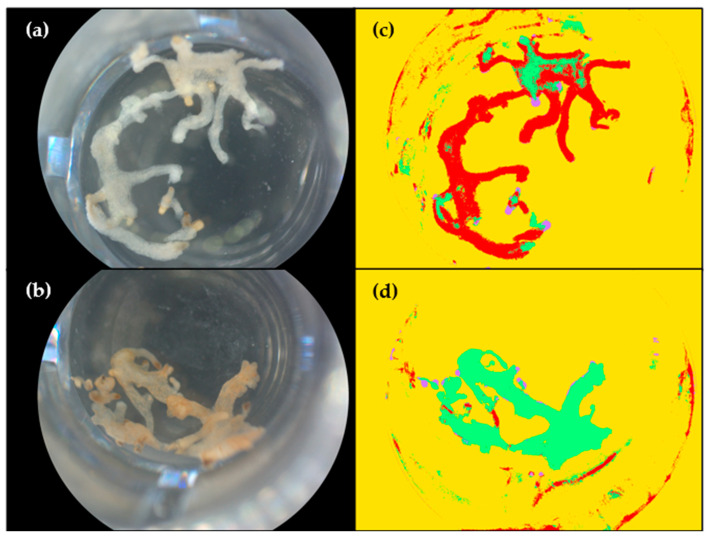
Example of the application of the necrosis analysis model on microcalli D6 after treatment with water or with fungal filtrates of modality A. (**a**,**b**) Images of microcalli treated with water (negative control) and filtrates of modality A (fungus–broomrape interaction) respectively after standardization step; (**c**,**d**) Images of the same wells after application of the classification model. Necrotic microcalli are shown in green, healthy ones are in red, seeds are in purple, and the background is in yellow.

The results of the image analysis are consistent with these observations, underlining the relevance of the method used (Figure 1). ANOVA on quasi-logistic regression showed a significant effect of filtrate (*p*-value < 2.2 × 10^−16^) and treatment time (*p*-value = 6.919 × 10^−12^) but not of the interaction between these two factors (*p*-value = 0.2326). Multiple comparisons for each factor (“filtrate” and “treatment time”) were performed. The rate of necrosis was significantly different between the filtrates (Table 1). For the “treatment time” factor, the only significant differences in the percentage of necrosis were observed between D0 and D5 (adjusted *p*-value = 5.57 × 10^−3^) and between D0 and D6 (adjusted *p*-value = 1.06 × 10^−3^).

**Figure 2:**
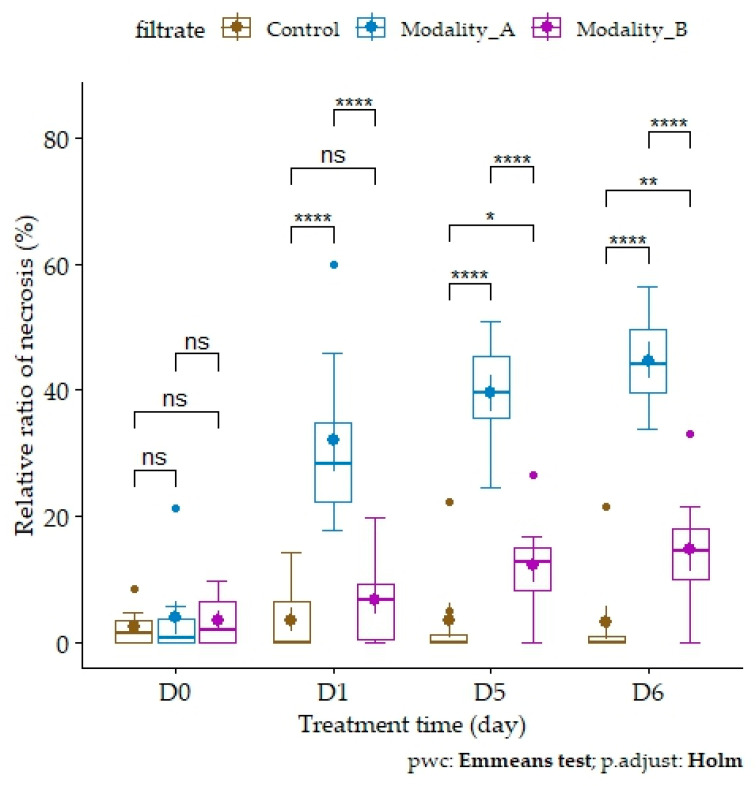
Necrosis development on broomrape microcalli depending on fungal filtrates over treatment time. The percentage of necrosis is the corrected relative ratio after subtraction of the average percentage of necrosis observed for the control at D0, D1, D5, and D6. Boxplots are shown with means (bold stars) and standard errors. Asterisks indicate significance of adjusted *p*-values (ns: not significant; *: 0.05; **: 0.01; ****: <0.001) after ANOVA on quasi-logistic regression post hoc pairwise comparison test between filtrates (Control: distilled water; modality A: filtrates from fungus–broomrape interaction; modality B: filtrates from fungus alone) for each day (Emmeans test with Holm–Bonferroni correction).

The analysis was further refined by pairwise comparisons between filtrates for each day (Figure 2). The average rate of necrosis in the control remained stable and low (<4%) over time. The progressive development of necrosis over time was confirmed for modalities A and B. Thus, the development of necrosis due to the absence of nutrients in the medium is minor, confirming the necrotic activity of fungal filtrates. More deeply, the necrosis increased after application of filtrates of modalities B and A (respectively, from 3.49 ± 4.13% to 14.7 ± 9.97% and from 3.84 ± 7.26% to 44.6 ± 8.16% from D0 to D6). The percentage of necrosis on microcalli treated with filtrates of modality A (32.0 ± 14.1%) was significantly different from the control (3.48 ± 5.33%) from D1. The necrotic activity of filtrates of modality B is later (significant difference with control from D5) and less intense than that of filtrates of modality A, which induced more necrosis more precociously (significant difference at D1, D5, and D6). As a result, fungus–broomrape interaction (modality A) leads to an increase in the exudation of necrotic compounds in the filtrates in terms of quality and quantity. 

### 2.2. Phytotoxic Fungal Metabolites Revealed by Untargeted Metabolomic Data Analysis

The initial databases obtained after metabolite extraction, UPLC-MS/MS data pretreatment, and manual metabolite annotation contained 3265 and 1964 features, respectively, in positive electrospray ionization (ESI+) and in negative electrospray ionization (ESI−). For the analysis then performed, manually annotated metabolites (AMs) and non-annotated metabolites (NAMs) were selected after the elimination of adducts and features with a relative standard deviation of quality controls of more than 25%. The analysis was based on 190 AMs and 1317 NAMs detected in ESI+, 52 AMs, and 1026 NAMs detected in ESI–. Relative abundance of the metabolites produced in the extracts from fungus–broomrape interaction (modality A), from fungus alone (modality B), and from broomrape alone (modality C) (four samples/modality) were analyzed by an ANOVA to distinguish statistically significant metabolites. A simple comparison of the normalized means and the heat map from ANOVA analysis highlighted the origin of the metabolite (from fungus or broomrape) and its level of accumulation during the interaction between fungus and parasitic plants.

#### 2.2.1. Results from the ESI+ Dataset 

**Figure 3:**
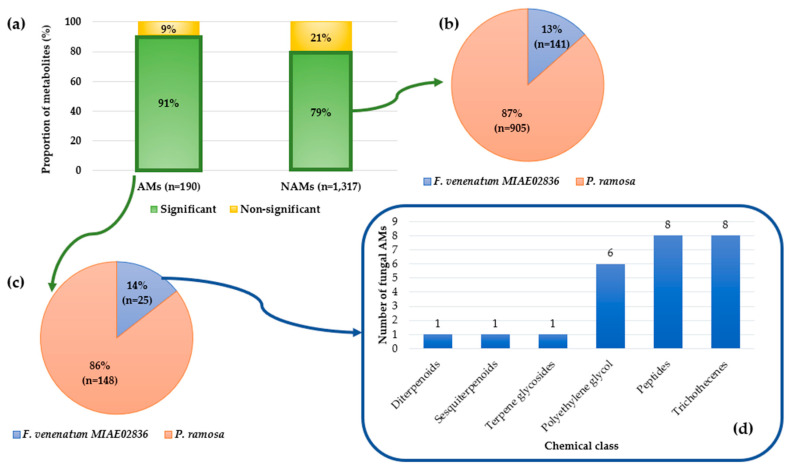
Results of metabolomic data analysis (ESI+ mode) for AMs and NAMs. (**a**) Proportion of metabolites with relative abundance significantly and non-significantly different based on ANOVA by permutation; (**b**,**c**) metabolites with relative abundance significantly different distributed according to their origin of production (*F. venenatum* MIAE02836 or *P. ramosa*) for NAMs and AMS respectively. The origin of production was supposed after a simple comparison between the normalized means of the relative abundance of the metabolites produced by the fungus alone (modality B) and by the broomrape alone (modality C); (**d**) distribution by chemical class of annotated fungal metabolites with relative abundance significantly different.

Statistical analysis performed using a one-factor ANOVA by permutation test highlighted a significant difference in the relative abundance of 173 AMs and 1046 NAMs between the three conditions. Among them, the major part has been identified as metabolites produced by *P. ramosa* (86% of the AMs and 87% of the NAMs) (Figure 3) after comparison between their normalized means from modality B (fungus alone) and modality C (broomrape alone). Among the NAMs, there was a statistical difference between modalities; 141 (13%) were identified as fungal in origin (Figure 3). The 25 AMs (14%) produced by *F. venenatum* MIAE02836 belong to six chemical classes, of which four are associated with the terpenoid family (diterpenoids, terpene glycosides, sesquiterpenoids, and trichothecenes). The majority of the fungal AMs are trichothecenes, peptides, and polyethylene glycol (PEG) (Figure 3).

**Figure 4:**
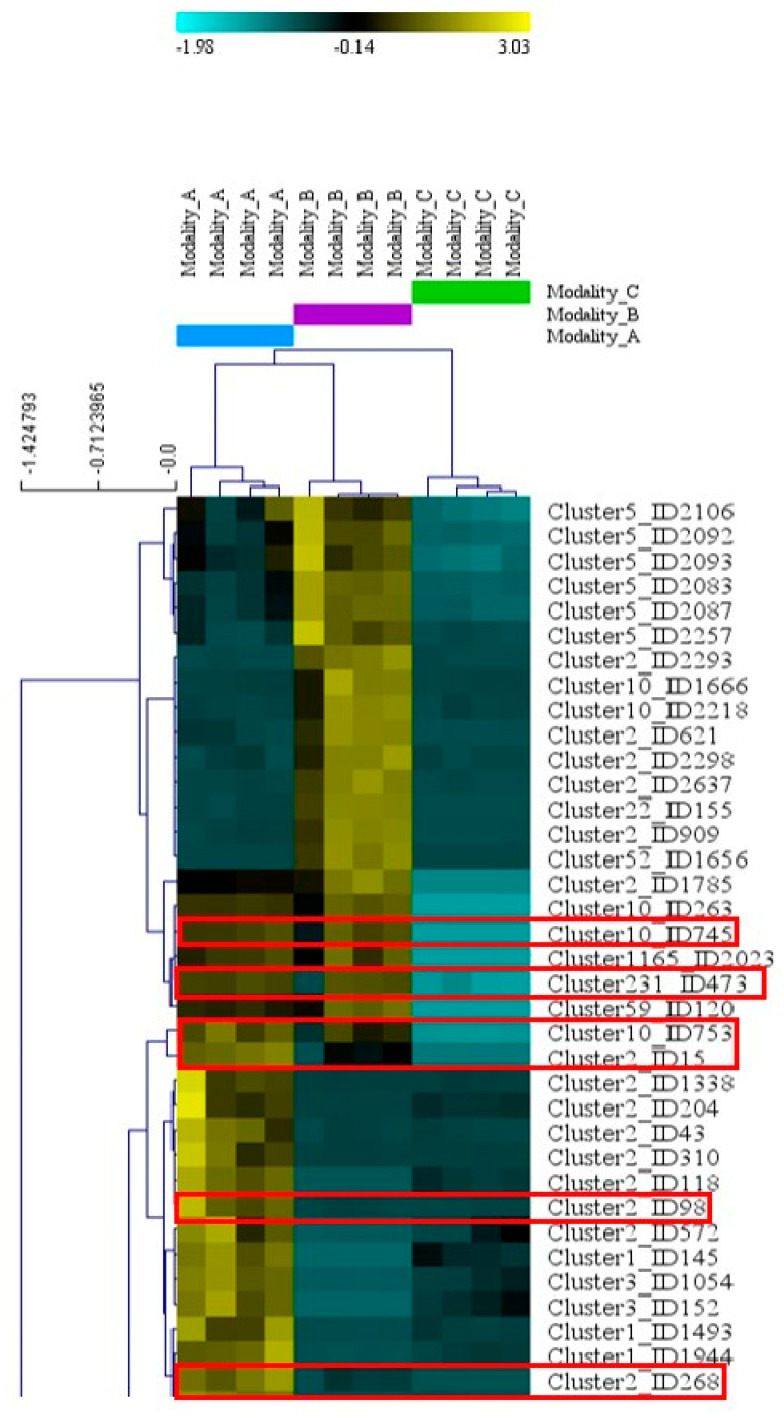
Accumulation of AMs with significantly different relative abundance by modality (broomrape alone, fungus alone, and interaction) (ESI+ mode). Part of the hierarchical tree showing metabolites with relative abundance significantly different between modalities A (broomrape–fungus interaction), B (fungus alone), and C (broomrape alone) according to the one-factor ANOVA by permutation (1000 permutations, *p*-value < 0.01) using MeV 4.9.0 software. The basal normalized relative abundance is shown in black. When the gradient tends towards yellow, the metabolite is accumulated to a greater extent. When the gradient tends towards blue, the quantity is lower. Metabolites framed in red correspond to fungal metabolites accumulated during interaction (see Table 2).

**Figure 5:**
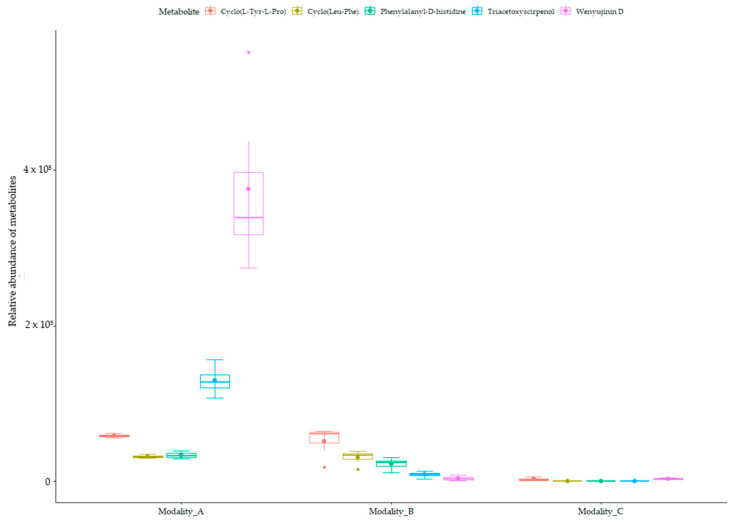
Mean relative abundance of fungal metabolites accumulated during the interaction with broomrape. Boxplots are shown with means (bold stars) and standard errors of the relative abundance of the fungal peptides and sesquiterpenoids accumulated during the interaction. Anguidin has not been represented, as it is heavily produced (minimum relative abundance of 329,458 metabolites and 2,442,767 metabolites for the fungus alone and for the interaction, respectively). Modality_A: filtrates from fungus–broomrape interaction; Modality_B: filtrates from fungus alone; Modality_C: filtrates from broomrape alone.

As a main result, among the 25 fungal AMs, three sesquiterpenoids and three peptides were overproduced during the interaction (modality A) compared to the two other modalities (B and C) (Table 2, Figure 4). The hierarchical tree of relative abundance of the analyzed metabolites shows that broomrape–fungus interaction (modality A) induces a more important accumulation of triacetoxyscirpenol, anguidine, and wenyujinin D compared to the fungus-alone (modality B). This difference in the level of production of the peptides is less pronounced. These results are confirmed by the mean relative abundance of the metabolites in extracts of the modalities A and B (Figure 5).

Among the 141 NAMs statistically different between modalities, 36 were overproduced during interaction (modality A). Comparing the clusters to which they belong with those of the AMs, only two NAMs (Cluster22_ID1801 and Cluster22_ID555) were attached to a common cluster, cluster 22. The fungal AM attached to this cluster is the diterpenoid gagunin E (Table 2). The metabolites Cluster22_ID1801 and Cluster22_ID555 may be diterpenoids. The other fungal NAMs accumulated during the interaction were distributed in 31 clusters for which no putative annotation could be made, hence the absence of hypotheses concerning their chemical class.

To go further in the characterization of the NAMs of interest, a search was performed with MS2Query. Among the 36 fungal NAMs overproduced during the interaction (modality A), six obtained a good analog prediction with a model score higher than 0.7, of which three were predicted exact matches with a precursor *m/z* difference below 1 Da (Table 3). For the two metabolites belonging to cluster 22 previously mentioned, model scores are in the range 0.6–0.7 (0.6337 for Cluster22_ID1801 and 0.6941 for Cluster22_ID555) with a high precursor *m*/*z* difference (68.0975 Da for Cluster22_ID1801 and 219.1209 Da for Cluster22_ID555) so the results obtained should be analyzed with caution. The natural product class predicted was one of cyclic peptides and depsipeptides, which could be produced by the amino acids and peptides or the polyketides pathway.

#### 2.2.2. Results from the ESI Dataset

Statistical analysis performed in the same way as for the ESI+ dataset using an ANOVA by permutation revealed a significant difference in the relative abundance of 50 AMs and 931 NAMs between the three conditions. Among them, 25 metabolites (1 AM and 24 NAMs) were supposed to be produced by the fungus after comparison between their normalized means from modality B (fungus alone) and modality C (broomrape alone). The highlighted fungal AM was also accumulated during the interaction with broomrape (modality A). This is the terpene glycoside abrisaponin A. Among the 24 NAMs identified as produced by *F. venenatum* MIAE02836, seven were overproduced during the interaction and belong to different clusters (clusters 2, 49, 257, 342, 576, 732, 825). No annotation was performed for the NAMs of these clusters except for those of cluster 2, which is associated with glycosylated flavonoids.

To overcome this issue, a new search using MS2Query was performed for the ESI+ dataset. Two analogs were found with a prediction score up to 0.7 for the NAMs Cluster_ID582 and Cluster_ID376 but were not exact matches (Table 4).

## 3. Discussion

### 3.1. Confirmation of a Phytotoxic Activity of Fungal Metabolites on Broomrape Microcalli

Phytotoxicity tests on microcalli using crude extracts of the fungus alone or in interaction with broomrape proved to be relevant, demonstrating phytotoxic activity of *F. venenatum* MIAE02836 metabolites towards *P. ramosa* microcalli. A browning and necrotic appearance of microcals exposed to fungal filtrates was observed as early as the first day post-application. Image analysis was successfully used to quantify the percentage of necrosis due solely to fungal filtrates, taking into account the natural dieback of microcalli in the absence of nutrients. Subtracting the average percentage of necrosis observed in the negative control (only water) produced results consistent with observations. Statistical analysis revealed a significant effect of filtrates on the appearance of necrosis compared with the control. After six days of treatment, fungus-alone (modality B) or fungus-broomrape interaction (modality A) filtrates induced 5 and 14 times more necrosis, respectively, than the control, suggesting the presence of bioactive fungal metabolites. Furthermore, the more important efficiency of fungus–broomrape (modality A) filtrates suggests the ability of broomrape to induce potential phytotoxic compounds. These results validate our first hypothesis and confirm the potential role of *F. venenatum* MIAE02836 as a biocontrol agent for *P. ramosa*. In addition to its dual ability to inhibit germination and cause necrosis on stems [11], this strain has also shown phytotoxic activity towards the early tissues of broomrape. The use of phytotoxic metabolites of *F. venenatum* MIAE02836 appears promising for targeting the early developmental stages of *P. ramosa* and thus reducing the soil seed bank, a major challenge in controlling this parasitic plant [35].

### 3.2. Specifications, Biaises, and Avenues for Improvement of Image Analysis in the Assessment of Fungal Filtrates Necrotic Activity

The image analysis method was functional in quantifying necrosis. With training on more than 400,000 pixels and taking into account the natural dieback of microcalli, the model was robust and revealed a phytotoxic effect of filtrates. However, one of the difficulties was photographing the wells using a ZEISS Axiocam ERc5s microscope camera connected to the associated ZEN 3.9 (ZEISS Efficient Navigation) microscopy software. This software enabled direct visualization of the wells, facilitating image capture (adjustment, focusing). Nevertheless, as the acquisition was manual, it was difficult to maintain homogeneity, particularly in terms of framing and brightness (differences in reflection depending on the position of the well on the plate and the mirror effect of calli on the well walls). A correction was made to highlight necrotic induction by fungal filtrates only. Optimizing image quality will enable us to quantify necrosis more accurately. In addition to image quality, the acquisition time parameter should not be overlooked. Manual photography is tedious and takes longer the larger the number of plates (and therefore wells) (around two hours to photograph four whole plates).

Despite these technical and time-consuming biases linked to the exploratory nature of our approach, the results obtained made it possible to validate our first hypothesis and then test the second one. They encourage continuing the approach by correcting these biases so as not to lose the advantages of phytotoxicity tests in 96-well plates (rapid production of microcalli, availability of a large number of samples). One possibility would be to automate image acquisition. Various systems adapted to 96-well plates have been developed based on microscope-based automated acquisition pipelines. An automated imaging system to facilitate sample screening in multi-well plates has already been designed [36]. The plate is set in motion via a motorized stage in all three dimensions, and Python-language scripts are used to scan and collect images from each well in an automated fashion. Built from a woodworking machine and a microscope-mounted camera, the tool has the advantage of being easily reproducible and inexpensive. Microscopy tools and software that enable automatic, customized digitization of plate well images, such as the Well Plate Navigator tool in the cellSens 5D image acquisition software, are available [37], as well as the JOBS module in the NIS-Elements software [38,39] or the ZEISS AI Sample Finder tool [40]. The latter also automates configuration parameters (sample placement, focusing, and experimental design) thanks to a deep neural network.

### 3.3. Identification of Fungal Phytotoxins Using Metabolomics Data Analysis

Statistical analyses using an ANOVA by permutation revealed metabolites with significantly different relative abundance between modalities. Fungal and broomrape metabolites were distinguished by comparing the normalized mean relative abundance of the metabolites from modality B (fungus alone) and from modality C (broomrape alone). Finally, the metabolites accumulated or not during the fungus–broomrape interaction (modality A) were identified. By cross-referencing these results (for ESI+ and ESI−), a total of 191 metabolites (26 AMs and 165 NAMs) were identified as being of fungal origin, 50 of which (7 AMs and 43 NAMs) were overproduced during the interaction with *P. ramosa*. The hypothesis, thus verified, was that these overproduced metabolites attacked metabolites of the fungus and were potentially deleterious to *P. ramosa*.

Of the seven fungal AMs accumulated during the fungus–broomrape interaction, three are peptides, including two diketopiperazines (cyclo(L-Tyr-L-Pro) and cyclo(Leu-Phe)) and one dipeptide (phenylalanyl-D-histidine). A diversity of fungal nitrogenous metabolites exists, and some of them have been recently reviewed for their phytotoxic activity against weeds [24]. Diketopiperazines, formed by the cyclization of two peptides, are known to be bioactive molecules, of which several, such as maculosin (cyclo(L-Tyr-L-Pro)), have shown phytotoxic activity [41]. Maculosin was identified as a fungal pathogen of *Zinnia elegans* by Kamikawa et al. [42]. Stierle et al. revealed the specificity of this phytotoxin produced by *Alternaria alternata* towards the weed *Centaurea maculosa* [43]. Production of this metabolite has also been demonstrated in other microorganisms, including fungi such as *Monascus pilosus* BCRC 38072 [44], *Alternaria raphani* [45], and *Aspergillus fumigatus* [46]. Although an emerging mycotoxin, maculosin has been characterized as non-toxic to porcine intestinal epithelial cells [47], hence its potential interest for biocontrol use. Cyclo(Leu-Phe) was isolated from filtrates of *Alternaria dauci*, the fungal pathogen that causes carrot leaf blight. It was identified with six other diketopiperazines in the most phytotoxic fractions, tested on detached leaves of parsley (*Petroselinum crispum*). Although no information was found concerning the phytotoxic potential of the dipeptide phenylalanyl-D-histidine, the phytotoxic activity of its two constituent amino acids has been highlighted. Among 13 amino acids tested for their germination-inhibiting activity on *P. ramosa* seeds, histidine completely inhibited germination at a concentration of 2 mM [48]. Phenylalanine and histidine strongly inhibited germination and radicule growth of the seeds of *Orobanche minor*, another parasitic weed [49]. Phenylalanine was also reported to be lethal to *P. ramosa* seeds. Applying the amino acid at a concentration of 2 mM resulted in a more than 90% decrease in germination accompanied by seed mortality of 94.07% [50].

The four other fungal AMs produced more in the presence of broomrape belong to terpenoids, of which three were sesquiterpenoids (wenyujinin D, triacetoxyscirpenol, and anguidine) and one terpene glycoside (abrisaponin A). To date, the production of abrisaponin A and wenyujinin D by fungi or their phytotoxicity has not been documented. These metabolites may have no herbicidal activity on *P. ramosa*, as their accumulation during interaction with the parasitic plant may be due to molecules, such as flavonoids, emitted by *P. ramosa*. The influence of flavonoids on the production of fungal secondary metabolites depends on the flavonoid (type, concentration, antiradical capacities, etc.) and the fungal strain [51]. For instance, the presence of apigenin in the medium led to an increase from 13.7 to 289.4% in total trichothecenes produced by *Fusarium culmorum* strain CBS 173.31. Triacetoxyscirpenol and anguidine (also known as diacetoxyscirpenol) are trichothecenes. Anguidine is particularly effective at inhibiting germination of *P. ramosa* seeds, reducing germination rates by over 90% at concentrations of 0.1 mM [52]. Trichothecenes, mainly produced by fungi of the *Fusarium* and *Trichoderma* genera, are nevertheless known to be toxic to mammals and other microorganisms [53,54,55].

A rather surprising result was the identification of fungal AMs belonging to the PEG class. Of the 25 fungal AMs, six are PEG. The manual annotation of the metabolite Cluster5_ID2092 (decaethylene glycol) was confirmed by MS2Query, predicting an exact match with a model score of 0.9242 (result not shown). The natural production of PEG by fungi is not mentioned in any scientific reference, suggesting possible contamination of extracts during chemical analysis by HPLC and MS. Another hypothesis would be that *F. venenatum* MIAE02836 does indeed produce PEGs, and the non-targeted metabolomics carried out highlighted this particularity. This hypothesis is supported by interesting information concerning ethylene. This gaseous unsaturated hydrocarbon forms the basis of numerous chemical reactions for the production of compounds such as PEG. Of major importance in the petrochemical industry, ethylene is produced by industrial synthesis from fossil fuels. However, this molecule was first discovered in plants at the beginning of the 20th century before being identified in several microorganisms, including fungi [56]. Twenty-five out of 86 pathogenic fungi showed their capacity to synthesize ethylene [57]. The species *F. oxysporum*, represented in particular by the forma speciales *vasinfectum* and *tulipae*, is one of the ethylene-producing species [57,58,59]. Various studies have also highlighted the impact of ethylene on plant growth and development. Ethylene produced by *Acremonium falciforme* reduced stem elongation, swelled the hypocotyl, and altered the growth direction of *Pisum sativum* var. *Alaska* [60]. Depending on ethylene concentration, the root growth of cereals and the nodulation capacity of legumes can be inhibited [61]. It is possible that *F. venenatum* MIAE02836 produces ethylene and then PEG, following a biosynthetic pathway not yet known, and that the PEG produced has phytotoxic activity on calli. This latter point remains to be demonstrated, especially as PEG are produced more by the fungus alone than during interaction with broomrape. Defense metabolites of *P. ramosa* may be involved in this reduction. The addition of L-methionine and riboflavin increased the ethylene production in *F. oxysporum* f. sp. *vasinfectum* but had a fungicidal effect on the fungus from 2.66 µM [58]. Riboflavin is one of the AMs identified as statistically different between modalities. It was produced by *P. ramosa* and accumulated during interaction with the fungus. The spores of *F. venenatum* MIAE02836 could have been affected by the presence of riboflavin, explaining a lower concentration of PEG in the extracts resulting from the interaction.

The majority of the NAMs accumulated during the interaction could not be attached to a known cluster and, therefore, to a chemical family. The 43 fungal NAMs overproduced during the interaction were distributed in 38 different clusters, i.e., potentially 38 different chemical families. To overcome this issue, a new spectra-based search was performed for these metabolites using MS2Query. Eight analogs were well predicted, with three of them being exact matches: Cluster129_ID49, Cluster283_ID586, and Cluster194_368 (Table 3).

The predicted exact match for Cluster129_ID49 is diacetoxyscirpenol, a well-known mycotoxin, as mentioned previously. Any other mycotoxin was clearly characterized, which is quite surprising as the *Fusarium* genus is known for the production of diverse fusariotoxins [62]. While *F. venenatum* has a reputation for being non-pathogenic and has been used for decades as a source of mycoproteins [14,15,16,17], this fungi is also able to produce type A trichothecenes such as butanolide, calonectrin, diacetoxyscirpenol or isotrichodermol [18,19,20,21]. In our study, the major part of detected metabolites remains unidentified, as mentioned above, which could explain the absence of these types of metabolites. Another explanation could be the cultural conditions that highly influence the production of mycotoxins by *Fusarium* [63,64,65]. In our study, metabolite production of *F. venenatum* MIAE02836 in the presence or not of broomrape was assessed in a minimal medium poor in nutrients and without glucose, at 25 °C for 24 h. It has been demonstrated that carbohydrate sources were favorable for mycotoxins production [66]. Temperature, water activity, or incubation time are also major parameters influencing fusariotoxins [63,67]. For instance, the optimum temperature for mycotoxins production by *F. graminearum*, a phylogenetically closely related fungi to *F. venenatum*, ranged from 24 to 28 °C [67]. It was also noticed that the production of deoxynivalenol and zearalenone by *F graminearum* was the highest on the 35th day of incubation [63].

Cluster283_ID586 was predicted to be a hydroxylated derivative of 14-bromodiscorhabdin C. The pyrroloiminoquinone alkaloid 14-bromodiscorhabdin C was fist isolated from a latrunculid sponge collected in the Tsitsikamma Marine Reserve (South-Africa), and as other marine alkaloids belonging to the discorhabdins, it has been associated with antimicrobial and anticancer activities [68,69,70]. No phytotoxicity of this compound and of other discorhabdins have been highlighted in the literature yet.

The Cluster194_368 predicted exact match is the pseudoguaiane sesquiterpenoids 1H-3a,6-Epoxyazulene-7-acetic acid, also known as ambrosic acid. This metabolite was discovered in the pollen of the invasive plant *Ambrosia artemisiifolia* collected in Japan and was first associated with hay fever [71]. More recently, ambrosic acid isolated from Korean *A. artemisiifolia* was characterized as a potential neuroprotective natural chemical against amyloid-β-induced cytotoxicity in Alzheimer’s disease [72]. Up to now, no phytotoxicity or other bioactivities have been documented for this compound, while several articles and reviews mentioned that pseudoguaiane sesquiterpenoids, and more generally sesquiterpenes lactones, exhibit broad-spectrum biological activities such as anti-carcinogenic, antimicrobial, anti-inflammatory, cytotoxic and phytotoxic activities among others [73,74,75,76]. Artemisinin from *Artemisia annua* [77], parthenin from *Parthenium hysterophorus* [78], costunolide, reynosin, and santamarine from *Bidens sulphurea* [79], or confertin, neoambrosin, and salsosol A and B from *Ambrosia Salsola* [73] are some examples of phytotoxic sesquiterpenes lactones promising for agricultural weed control.

Further metabolite annotation using molecular networks and complementary machine-learning tools such as MS2Query [80], Sirius [81], or TIMA (Taxonomically Informed Metabolite Annotation) [82] is required to characterize fungal NAMs, which were far more numerous and diversified than AMs. Further identification opens up prospects for the discovery of new molecules and the deepening of knowledge in fungal metabolomics, which is still in its infancy [83].

Thus, although questions remain as to the nature and functions of the metabolites, analysis of the data has identified phytotoxic fungal metabolites. Among the fungal metabolites accumulated during the interaction, four are known to have herbicidal activity: maculosin, cyclo(Leu-Phe), phenylalanyl-D-histidine, and anguidine. This confirms the results of phytotoxicity tests, which showed a significant effect of crude fungal extracts on *P. ramosa* microcalli, particularly those resulting from the interaction between broomrape and *F. venenatum* MIAE02836. The next steps would be to pursue the bioassay-guides isolation of herbicidal compounds from *F. venenatum* MIAE02836 using fractions of the crude extracts and even several solvents of increasing polarity in order to optimize extraction and refine the analysis.

## 4. Conclusions

The framework of this study was the search for phytotoxic fungal metabolites for *P. ramosa* using *F. venenatum* strain MIAE2836, isolated from symptomatic branched broomrapes and identified as a promising candidate for *P. ramosa* biocontrol. Phytotoxicity tests of fungus–broomrape interaction crude extracts on *P. ramosa* microcalli confirmed the phytotoxic potential of *F. venenatum* MIAE02836 towards the early developmental stages of the parasitic plant. The methodology used proved to be robust. The qualitative observation of necrosis on microcalli treated with fungal filtrates was confirmed quantitatively by image analysis. Analysis of non-targeted metabolomics data revealed metabolites produced by *F. venenatum* MIAE02836. The choice of fungal metabolites overproduced during interaction with broomrape was relevant. Maculosin, cyclo(Leu-Phe), phenylalanyl-D-histidine, and anguidine identified in this way are known to be phytotoxic to various plants, including parasitic plants such as *P. ramosa*. However, the majority of fungal metabolites have not yet been identified. Fungal metabolites accumulated during the interaction but not yet annotated are six times more numerous than those identified and are distributed in a greater number of clusters, suggesting a molecular diversity. This initial exploratory work validates our two hypotheses and achieves our first objective. It is the first step needed for acquiring fundamental knowledge of plant–pathogen interactions and opens up interesting prospects for the use of fungal metabolites to control *P. ramosa*.

## 5. Materials and Methods

### 5.1. Plant Material

#### 5.1.1. Production of *Phelipanche ramosa* Microcalli

Seeds of *P. ramosa* were collected from mature floral capsules from highly infested oilseed rape fields at Echiré (46°22′49″ N, 0°27′24″ W) in western France in 2019. The genotype of the seed batch belongs to the “genetic cluster 1”, primarily capable of infesting oilseed rape [6]. Collected seeds were sieved and then kept in watertight glass containers at ~20 °C. Microcalli of *P. ramosa* were prepared according to previous protocols [30,31,84] as follows.

First, seeds were surface-disinfected in vigorous agitation in a 2.4% sodium hypochlorite (Na(OCl)_2_) solution for 5 min and then rinsed with sterile deionized water three times for 1 min and three times for 5 min. Seeds were suspended (10 mg seeds. mL^−1^) in HEPES (HEPES, Sigma-Aldrich) buffer (1 mM; pH 7,5 adjusted with KOH) sterilized by syringe filtration 0.2 µm (Disposable filter 30 mm AC 0.2 µm, ClearLine, Dutscher, Bernolsheim, France). Seeds were kept in the dark at 21 °C for 7 days for the conditioning period [85].

Then, to initiate germination, conditioned seeds were rinsed three times with sterile distilled water and resuspended (2.5 mg seeds mL^−1^) in the HEPES buffer supplemented with the germination stimulant *rac*-GR24 at 10^−7^ M provided by Dr. Binne Zwanenburg (Radboud University, Netherlands). Fifty microliters of this suspension were then distributed in each well of 96-well plates (Cell Culture Microplate 96-Well, Cellstar^®^, Greiner Bio-One, Kremsmünster, Austria). Plates were placed at 21 °C in the dark in an air-conditioned module. After 4 days, germination was checked under a stereoscopic microscope (1.95-250x, Carl Zeiss Stemi 2000-C, 444036-9000, Carl Zeiss Microscopy, New York, NY, USA). When seeds did not germinate, the plates were placed back in a climate-controlled module until the radicule protruded.

To allow the development of microcalli, the germination medium was replaced with 100 µL of MS culture medium [86] including Nitsch vitamins (Murashige & Skoog medium including Nitsch vitamins, Duchefa Biochemie) supplemented with MES (MES, Sigma-Aldrich, St. Louis, MO, USA) buffer (0.49% *w*/*v*) and saccharose (2% *w*/*v*) (pH 5.75 adjusted with KOH) one day after seed germination. Plates were kept at 21 °C in the dark, and the culture medium was renewed weekly.

#### 5.1.2. Production of *Phelipanche ramosa* Floral Stems

Seeds of a population of *P. ramosa* parasitizing tobacco (*Nicotiana tabacum* L.) were collected from mature floral capsules in highly infested tobacco arable lands at Aigre (45°56′41.363″ N, 0°31′3.947″ W) in western France in 2017. The genotype of the seed batch belongs to the “genetic group x”, which characterizes populations able to infest tobacco and tomato [6]. Collected seeds were sifted and then kept in watertight glass containers at −20 °C. Tobacco seeds of the ITB683 variety were kindly provided by L. Gatard (Coopérative Tabac Feuilles de France, Strasbourg, France). The stems of *P. ramosa* of genetic group x were produced from tobacco cultivated in pots under controlled conditions adapted from [2] with slight modifications. Several 5 L pots were filled successively with the following autoclaved layers:

- A layer of absorbent paper to prevent the substrate from escaping through the holes at the base of the pot;
- A layer of clay pebbles and a layer of pozzolan to drain;
- A layer of substrate, which is a mixture of 1/3 sand and 2/3 soil (57% silt, 35% clay, 8% sand) provided by the INRAE Experimental Unit of Epoisses (47°14′26″ N, 05°06′51″ E; Côte d’Or, France) supplemented with pozzolan;
- A layer of this same substrate supplemented with seeds of *P. ramosa*.

Two drippers per pot were added, distributing 2 L water/day each. All the pots were kept in the dark at 20 °C for 2 weeks to allow preconditioning of the parasite seeds. After this period, tobacco seeds were sown. Co-culture was assayed until emerging *P. ramosa* floral stems were sufficiently developed. Stems were regularly cut and then stored at −20 °C until their use.

### 5.2. Fungal Growth and Spore Production

*Fusarium venenatum* MIAE02836 was isolated from symptomatic *P. ramosa* associated with oilseed rape pathovar, genetic cluster 1 [11]. It showed dual competence, i.e., germination inhibition and necrotic activity, without being pathogenic to the tested host plants (tobacco and oilseed rape) [11]. The strain is cryopreserved at −80 °C in the Microorganisms of Interest for Agriculture and Environment (MIAE) collection (INRAE, Dijon, France).

The strain was grown in a tube on an agar slant using Potato Dextrose Agar (PDA, 39 g L^−1^) for 7 days. Three milliliters of autoclaved Malt Extract Broth (MEB 10 g L^−1^ malt) were added on top of the agar slant. Then, the culture surface was scraped with a sterile oese, and spores were suspended in a vortex. The fungal suspension obtained was added to 250 mL of autoclaved MEB and then put in an incubator (INFORS) under agitation (125 rpm) at 25 °C for five days to allow fungal growth.

The fungal culture was filtrated through a funnel fitted with a glass fiber filter (40–100 µm), collected in 50 mL Falcon tubes, and centrifuged at 7000× *g* for 5 min. The supernatant was discarded, and pellets were resuspended in Glucose-free Minimal Medium (GMM; NaNO_3_ 2g L^−1^, KH_2_PO_4_ 1g L^−1^, MgSO_4_-7H_2_O 0.5 g L^−1^, KCl 0.5 g L^−1^ and trace element solution 2 mL) and pooled into a single falcon tube. The trace element solution used for GMM contained citric acid 5 g L^−1^, ZnSO_4_-7H_2_O_5_ g L^−1^, FeSO4-7H2O 4.75 g L^−1^, Fe(NH_4_)_2_-(SO_4_)_2_–6H_2_O 1 g L^−1^, CuSO_4_-5H_2_O 0.25 g L^−1^, MnSO_4_-H_2_O 50 mg L^−1^, H_3_BO_4_ 50 mg L^−1^ and NaMoO_4_-2H_2_O50 mg L^−1^. Spores were quantified using Malassez cells, and fungal suspension volume was adjusted to 10^5^ spores mL^−1^.

### 5.3. Preparation of Crude Extracts for Phytotoxicity Essays and Metabolomics Analysis

To study the metabolite production of the fungus, the strain MIAE02836 was placed in co-culture with stems of *P. ramosa* or alone.

For the co-culture, a broomrape medium was prepared by mixing broomrape stems with GMM. First, stems of *P. ramosa* were surface-disinfected by a 90 s soak in 70% ethanol, then rinsed by three successive 30 s soaks in sterile osmotic water and were mixed with GMM (0.1 g stem/mL).

Then, three types of 20 mL crude extracts were prepared and replicated four times:

- Fungal suspension (10^5^ spores mL^−1^) with broomrape medium for the broomrape–fungus interaction (modality A);
- Fungal suspension in GMM (10^5^ spores mL^−1^) for strain only (modality B);
- Broomrape medium as a negative control (modality C).

Additionally, the GMM medium alone was tested as a blank.

All crude extracts were kept in the INFORS incubator under agitation (125 rpm) at 25 °C. After one day, each extract was centrifuged at 7000× *g* for 5 min. The supernatant was filtered through a 0.2 µm filter (Disposable filter 30mm AC 0.2 µm, ClearLine) and stored at −20 °C until lyophilization. Each lyophilized extract was used for both the phytotoxicity test and the metabolomics analysis.

### 5.4. Analysis of Necrotic Induction by Fungal Metabolites on Phelipanche ramosa Microcalli

#### 5.4.1. Methodological Development

To quantify the necrotic activity of the fungal filtrates on microcalli, we developed a machine learning-based workflow that uses pixel classification to automatically measure necrosis in branched broomrape tissues. Image analysis was conducted using Fiji, an open-source image processing software based on ImageJ (https://imagej.net/software/fiji/; [33]), combined with the *Trainable Weka Segmentation plugin* [87]. This approach enables the distinguishment of pixels corresponding to healthy and necrotic tissues, allowing for the calculation of the necrotic ratio.

**Figure 6:**
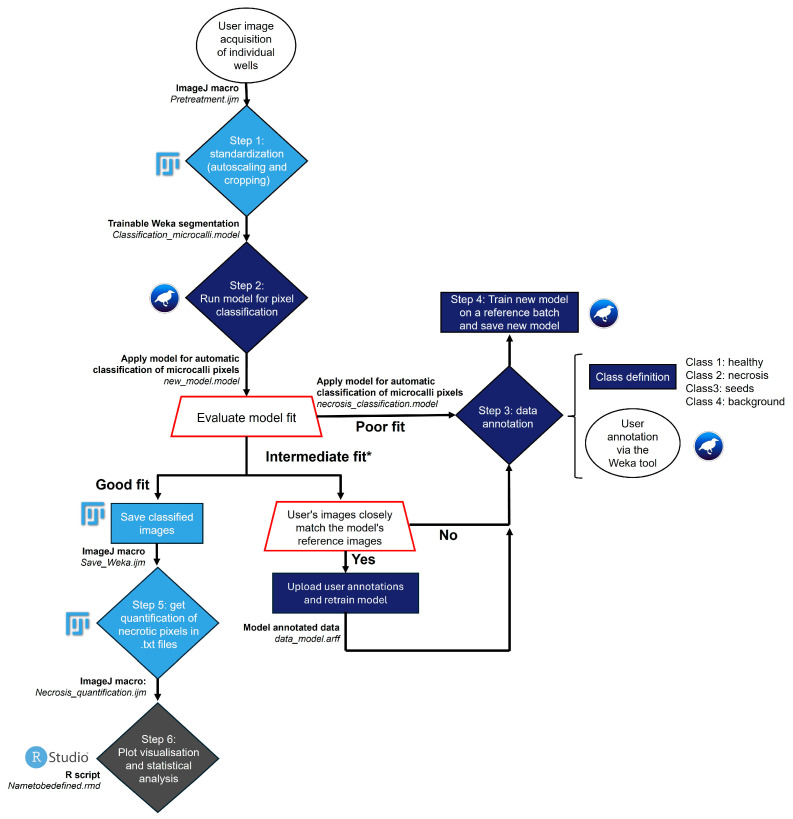
Workflow Overview for BRoomrape’s microcalli Automated Image-based Necrosis-detection (BRAIN). This workflow supports batch processing of images following a standardization step (*Step 1*) where users can choose to (i) run our pre-trained model on broomrape microcalli images (*Step 2; requires same image acquisition parameters*), (ii) annotate their own images (*Step 3*) and train a new model (*Step 4*), or (iii) annotate their own images and further train the pre-trained model (*Alternative Step 3 and Step 4*).

The workflow, BRoomrape’s microcalli Automated Image-based Necrosis-detection (BRAIN), is available on GitHub at https://gitlab.univ-nantes.fr/rhizoplante_experiments_r_registery/brain. It includes a comprehensive guide on the methodology, materials, and a dataset with all necessary files for the workflow, including Fiji macro and R scripts, pre-trained models, and a practice dataset. The procedure is summarized in the flowchart (Figure 6).

#### 5.4.2. Phytotoxicity Assays: Analysis to Evaluate Necrotic Activity

For the phytotoxicity assay, the crude filtrates of modalities A and B were tested on broomrape microcalli. Lyophilized filtrates were rehydrated with sterile distilled water and filtered at 0.2 µm (Disposable filter 30 mm AC 0.2 µm, ClearLine) to address the potential presence of solid residues and only test metabolite activity.

**Figure 7:**
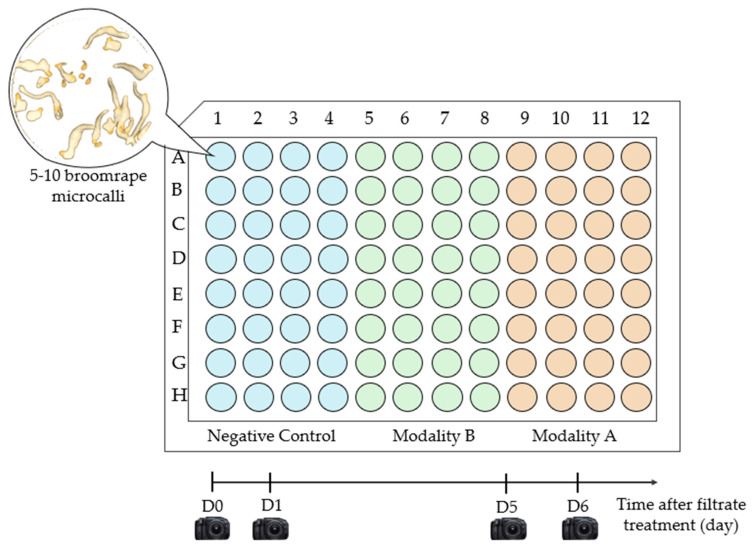
Sampling plan for phytotoxicity test. Columns 1–4 correspond to the negative control (microcalli with distilled water); columns 5–8 correspond to the filtrates of modality B (fungus alone); columns 9–12 correspond to the filtrates of modality A (fungus–broomrape interaction).

Necrotic activity was evaluated on one plate of one-month-old *P. ramosa* microcalli containing 5 to 10 microcalli per well. The culture medium was replaced by 50 µL of the filtrates (A or B) and 50 µL of sterile distilled water. As a negative control, microcalli were also treated with sterile distilled water. Thirty-two wells were used per modality (Figure 7). Necrosis induction was monitored qualitatively under a stereoscopic microscope (1.95-250x, Carl Zeiss Stemi 2000-C, 444036-9000) for six days (D1 to D6), and photographs were taken with a ZEISS Axiocam ERc5s camera at D0 (first day of the treatment), D1, D5, and D6 (Figure 7).

According to the image analysis methodology described below (Section 5.4.1; Figure 6), the photographs were first standardized. In order to process more data more quickly, the size of the images (in pixels) was reduced. Then, the necrosis recognition model on *P. ramosa* calli was trained on 30 images (10 per modality). The segmentation work was optimized by annotating pixels on stacks of 10 images rather than frame by frame. A total of 408,114 pixels were annotated. Then, the model developed was applied to image stacks to assess the necrosis rate of microcalli. A total of 96 images from twenty-four wells (eight per modality) taken at D0, D1, D5, and D6 were used for analysis. The number of pixels belonging to each class was calculated for each segmented image. The percentage of necrosis was then calculated for each day. To take into account the natural development of necrosis due to the absence of nutrients, the relative necrosis ratio was corrected by subtracting the average necrosis percentage obtained for the control.

#### 5.4.3. Statistical Analysis

Statistical analysis was performed using R 4.3.0 software (https://www.r-project.org/ (accessed on 27 July 2023); [88]). The effects of the treatment time (D0, D1, D5, and D6 after the addition of the filtrates) and the modalities of filtrates (A or B) on the relative necrosis ratios were analyzed via a generalized linear model (GLM) based on a “quasi-logistic” regression (a quasi-binomial distribution with the logit link function). The significance of the results was confirmed using the χ^2^ test [89]. Multiple comparisons of means were performed using the emmeans package [90] with Holm–Bonferroni *p*-value adjustment.

### 5.5. Untargeted Metabolomics Analysis for the Identification of Phytotoxic Fungal Metabolites

#### 5.5.1. Metabolite Extraction and UPLC-MS/MS Analysis

Lyophilized samples were resuspended in acetonitrile (1.5 mL) and water (1 mL), shaken before the addition of 2 g of MgSO_4_/NaCl salt mixture (4/1 *w*/*w*) and shaken again. Then, they were centrifuged at 5000× *g* for 10 min to collect the acetonitrile phase. Acetonitrile extracts were evaporated under reduced pressure, resuspended in 250 µL 10% acetonitrile in water, and filtered with glass microfiber filters (Cat. NO. 1820-037, Whatman International Ltd., Little Chalfont, UK) and distributed in HPLC vials.

Untargeted analysis was performed using a UHPLC system (Ultimate 3000 Thermo) coupled to a quadrupole time of flight mass spectrometer (Q-Tof Impact II Bruker Daltonics, Bruker, Billerica, MA, USA). A Nucleoshell RP 18 plus reversed-phase column (2 × 100 mm, 2.7 µm; Macherey-Nagel) was used for chromatographic separation, with a flow rate of 0.4 mL.min^−1^ for 5 µL injected. The mobile phases used were (A) 0.1% formic acid in water and (B) 0.1% formic acid in acetonitrile. The following gradient was used: 95% A for 1 min, followed by a linear gradient from 95% A to 80% A from 1 to 3 min, then a linear gradient from 80% A to 75% A from 3 to 8 min, a linear gradient from 75% A to 40% A from 8 to 20 min; then, 0% of A was held until 24 min, followed by a linear gradient from 0% A to 95% A from 24 to 27 min. Finally, the column was washed by 30% A for 3.5 min, then re-equilibrated for 3.5 min (35 min total run time).

Data-dependent acquisition methods were used for mass spectrometer data in positive and negative electrospray ionization (ESI) modes using the following parameters: capillary voltage, 4.5 kV; nebulizer gas flow, 2.1 bar; dry gas flow, 6 L.min^−1^; drying gas in the heated electrospray source temperature, 200 °C. Samples were analyzed at 8 Hz with a mass range of 100–1500 *m/z*. Stepping acquisition parameters were created to improve the fragmentation profile with a collision radio frequency (RF) from 200 to 700-volt peak-to-peak (Vpp), a transfer time from 20 to 70 µsec, and collision energy from 20 to 40 eV. Each cycle included an MS full scan and 5 MS/MS collision-induced dissociation (CID) on the 5 main ions of the previous MS spectrum.

#### 5.5.2. UPLC-MS/MS Data Pretreatment

Raw data were converted to .mzXML format using the MSConvert software (ProteoWizard package 3.0; [91]) before being processed using MZmine 2.52 software (https://mzmine.github.io/download.html (accessed on 02 November 2021); [92]) for both positive and negative data files.

The parameters were adjusted as follows: mass detection was performed using the centroid mass detector with the noise level set to 1.0 × 10^3^ for MS1 and 0 for MS2. The ADAP chromatogram builder [93] was used to generate the chromatogram profile with a minimum group size of scan 5, a group intensity threshold of 1.0 × 10^3^, a minimum intensity of 1.0 × 10^3^, and *m*/*z* tolerance of 10 ppm. The ADAP wavelets algorithm was used for chromatogram deconvolution. MS/MS scans were paired using a *m*/*z* tolerance range of 0.05 Da and a retention time (RT) tolerance of 0.5 min. The intensity window S/N was used as an S/N estimator with an S/N threshold of 10, a minimum peak height of 1.0 × 10^3^, a coefficient area threshold of 40, a peak duration range from 0.01 to 1 min, and an RT wavelet range from 0.02 to 0.2 min. Isotopes were detected using the isotopic peak grouper algorithm with an *m*/*z* tolerance of 10 ppm, an RT tolerance of 0.2 min, the maximum isotope set at 2, and the representative isotope used was the most intense. All the peaks were filtered using the feature list row filter, which kept only the peaks with the MS2 scan. The samples were aligned using the join aligner with an *m*/*z* tolerance of 10 ppm, a weight of *m*/*z*, an RT of 1, and an RT tolerance of 0.2 min. Then, blank subtraction, gap filling, the removal of duplicates, and manual validation of chromatographic peaks were successively performed. Metacorrelate was used to perform feature grouping with an RT tolerance of 0.2 min 0.005 Da; for the adduct search, an ion identity network was used with an *m*/*z* tolerance of 10 ppm.

#### 5.5.3. Molecular Network Generation and Manual Annotation

Processed data exported in .mgf and .csv files from MZmine analysis were used in MetGem 1.3.0 software (https://metgem.github.io/ (accessed on 4 November 2021); [94]) to build molecular networks. These later were generated with a cosine score (CS) threshold of 0.65. Metabolite annotation was performed in four steps. Data used for molecular networks were then searched against the available MS^2^ spectral libraries (GNPS Sigma’s Mass Spectrometry Metabolite, NIH natural Products, NIST14 Tandem, Massbank (NA) Spectral) with absolute *m*/*z* tolerance of 0.02, 4 minimum matched peaks, and minimal CS of 0.65. A manual annotation was also performed by matching fragmentation spectra to reference data of each feature with our homemade library (IJPB Plant Observatory-Chemistry, metabolism platform) and online databases. Non-annotated metabolites belonging to molecular network clusters containing annotated metabolites from the two previous steps were assigned to the same chemical family. After these steps, both ESI+ and ESI– datasets contained annotated metabolites (AMs), i.e., metabolites with putative name and/or chemical class, and not-annotated metabolites (NAMs), i.e., metabolites without putative name and/or chemical class.

#### 5.5.4. Statistical Analysis of Untargeted Metabolomic Data

The analysis of the datasets was performed with AMs and NAMs after the elimination of adducts and also features with a relative standard deviation of quality controls of more than 25%. The aim was to perform sorting by keeping the metabolites that are statistically different between test modalities A, B, and C and then identifying the origin of the metabolites on the basis of the relative abundance means of these metabolites. Particular attention was paid to metabolites that were supposed to be produced by the fungus and accumulated in extracts resulting from the interaction between the fungus and the parasitic plant, their overproduction probably being a sign of harmful activity towards *P. ramosa*. To achieve this, a one-factor permutation ANOVA (1000 permutations, *p*-value < 0.01) was first performed using MeV 4.9.0 software [95] to eliminate metabolites without relative abundance significantly different between modalities A, B, and C. A hierarchical tree was also built using Pearson correlation as a distance metric to visualize significant metabolites. Then, normalized means were compared using SI.CONDITIONS function in Excel to detect the origin of the metabolites (from broomrape or fungus) and their level of accumulation (overproduced or not during interaction) (Table 5). The results were compared with the heat map, and histograms of metabolite intensities versus extracts were plotted to refine interpretation.

#### 5.5.5. Further Annotation of Non-Annotated Metabolites of Interest

According to the previous results, a spectra-based analog search was performed for significant fungal metabolites with no or unclear annotation using MS2Query (https://github.com/iomega/ms2query (accessed on 1 August 2024); [80]). MS2Query is a machine-learning-based tool using MS2 mass spectral data to detect potential analogs and exact matches. It combines Spec2Vec and MS2Deepscore, two chemical similarity predictors outperforming the usually used cosine-based scores [80,96,97].

Analogs and exact matches (i.e., a precursor *m*/*z* difference < 1 Da) with an MS2Query score above 0.7 (score threshold above which many good analogs and exact matches are predicted) were considered to further annotate the fungal NAMs. The outputs from molecular networking, statistical analysis, and analog search were combined and visualized using Cytoscape 3.10.2 software [98].

## Figures and Tables

**Table 1:** Results of Emmans test to compare necrosis percentage induced by filtrates. Post hoc multiple comparisons based on ANOVA on quasi-logistic regression were performed using the Emmans test. Results are averaged over the levels of the “treatment time” factor. *p*-value was adjusted using the Holm–Bonferroni method for three tests. Asterisks indicate the significance of adjusted *p*-values ((*): 0.05; (****): <0.001).

Factor	Response Variable	Group 1	Group 2	*p*-Value Adjusted
**filtrate**	necrosis	Modality B	Control	4.62 × 10^−2^ (*)
		Modality B	Modality A	2.37 × 10^−9^ (****)
		Control	Modality A	2.31 × 10^−13^ (****)

**Table 2:** AMs having significantly different relative abundance and identified as produced by *F. venenatum* MIAE02836. Metabolites are classified according to their chemical class and presented by their manual annotation. The third column shows the cluster to which each metabolite is attached, followed by a unique identifier generated during data pre-processing. Metabolites in bold and italic are those accumulated during interaction with broomrape.

Chemical Class	Manual Annotation	Cluster_ID
**Sesquiterpenoids (trichothecenes)**	***Triacetoxyscirpenol***	***2_268***
	***Anguidin***	***2_15***
	7,8-Dihydroxydiacétoxyscirpenol	2_2293
	Xrusrvhnyvokli-vchtzwmcsa-	2_1785
	Xrusrvhnyvokli-vchtzwmcsa- isomer	2_2637
	3,15-Diacetyldeoxynivalenol	2_909
	Erigerolide	2_261
	[4-Acetyloxy-3-hydroxy-2-(hydroxymethyl)-1,5-dimethylspiro[8-oxatricyclo[7.2.1.02,7]dodecane-12,2′-oxirane]-10-yl] acetate	2_2298
**Peptides**	***Phenylalanyl-D-histidine***	***10_753***
	***Cyclo(Leu-Phe)***	***10_745***
	***Cyclo(L-Tyr-L-Pro)***	***231_473***
	PyroGlu-Ile	52_1656
	Cyclo(L-Leu-L-Pro)	59_120
	Phenylalanyl-proline	10_263
	PyroGlu-Tyr	10_2218
	PyroGlu-Phe	10_1666
**Polyethylene glycol**	Nonethylene glycol	5_2087
	Octaethylene glycol	5_2083
	HO-PEG5-CH_2_COOH	5_2257
	Decaethylene glycol	5_2092
	Undecaethylene glycol	5_2093
	Dodecaéthylene glycol	5_2106
**Terpene glycosides**	Soyasaponine I	1165_155
**Sesquiterpenoids**	***Wenyujinin D***	***2_98***
**Diterpenoids**	Gagunin E	22_155

**Table 3:** Predicted analogs and exact matches of significant fungal NAMs overproduced in the interaction using MS2Query (ESI+ dataset). The first column shows the cluster to which each metabolite is attached, followed by a unique identifier. The following columns show the predicted analogs with the natural product class associated, the model score prediction, and the precursor *m*/*z* difference obtained from MS2Query. Predicted exact matches are in bold.

Cluster_ID	Predicted Analogue and Natural Product Class	Model Prediction	*m/z* Difference
**192_365**	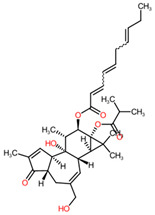 12b-O-[deca-2Z,4E,7Z-trienoyl]-13_-isobutyroyloxy-4b-deoxyphorbol(Tigliane diterpenoids)	0.7138	158.1498 Da
**1022_1830**	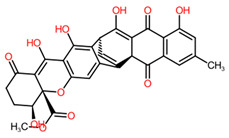 Xanthoquinodin A2_130147 (Anthraquinones and anthrones)	0.7171	13.9423 Da
**269_554**	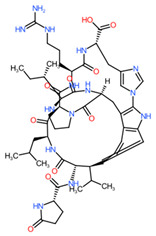 Amaranthipeptide A (Cyclic peptides)	0.7335	215.2626 Da
**129_49**	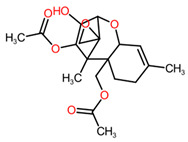 Diacetoxyscirpenol (Trichothecane sesquiterpenoids)	**0.7364**	**5 × 10^−4^ Da**
**283_586**	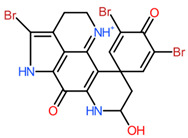 Hydroxylated 14-bromodiscorhabdin C (+2)	**0.7907**	**0.8294 Da**
**194_368**	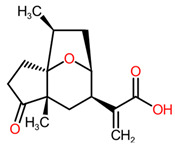 1H-3a,6-Epoxyazulene-7-acetic acid (Pseudoguaiane sesquiterpenoids)	**0.8197**	**1 × 10^−4^ Da**

**Table 4:** Predicted analogs of significant fungal NAMs overproduced in the interaction using MS2Query (ESI− dataset). The first column shows the cluster to which each metabolite is attached, followed by a unique identifier. The following columns show the predicted analog with their chemical subclasses, the natural product class associated, the model score prediction, and the precursor *m*/*z* difference obtained from MS2Query.

Cluster_ID	Predicted Analogue and Chemical Subclass	Model Prediction	*m/z* Difference
**342_582**	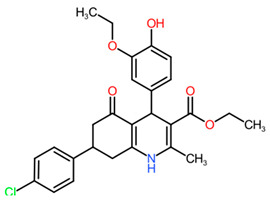 Ethyl 7-(4-chlorophenyl)-4-(3-ethoxy-4-hydroxyphenyl)-2-methyl-5-oxo-1,4,6,7,8 -pentahydroquinoline-3-carboxylate M+ formate (Phenylquinolines)	0.7371	28.8941 Da
**2_376**	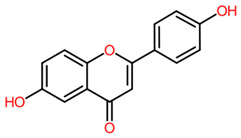 6,4′-Dihydroxyflavone (Flavones)	0.8322	79.9564 Da

**Table 5:** Formula used in Excel to characterize the production of the metabolites. P_x_, Q_x_ et R_x_ are the means (normalized on MeV) of the relative abundance of the metabolite x for the modalities A (interaction broomrape–fungus), B (fungus alone), and C (broomrape alone), respectively.

Production of the Metabolite	Formula Used
**Broomrape or fungus**	=SI.CONDITIONS(Q_x_ < R_x_; “Broomrape metabolite”; Q_x_ > R_x_; “Fungal metabolite”)
**Accumulation in modality A vs. modality C**	=SI.CONDITIONS(P_x_ < R_x_; “SIGNAL DOWN in Interaction”; P_x_ > R_x_; “SIGNAL UP in Interaction”)
**Accumulation in modality A vs. modality B**	=SI.CONDITIONS(P_x_ < Q_x_; “SIGNAL DOWN in Interaction”; P_x_ > Q_x_; “SIGNAL UP in Interaction”)

## Data Availability

The comprehensive guide on the BRoomrape’s microcalli Automated Image-based Necrosis-detection (BRAIN) workflow developed in this study, including methodology, materials, and a dataset with all necessary files (Fiji macro and R scripts, pre-trained models, and a practice dataset) is available at https://gitlab.univ-nantes.fr/rhizoplante_experiments_r_registery/brain.
